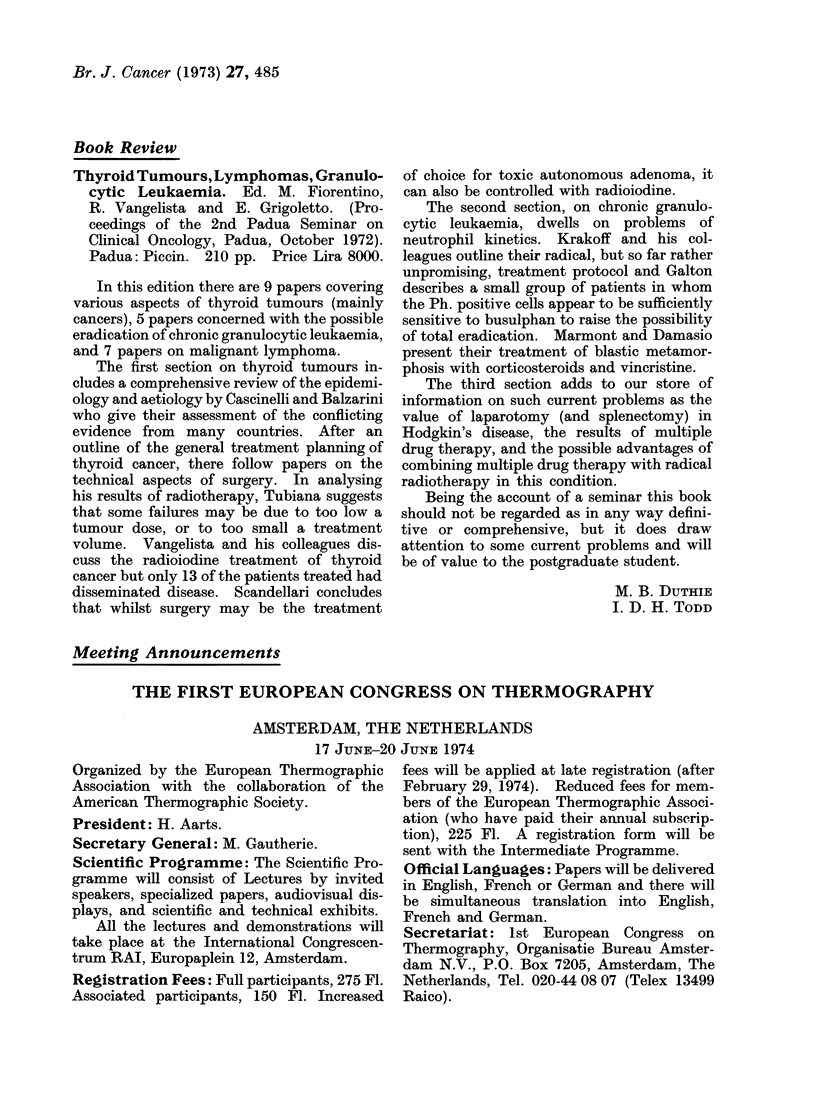# Thyroid Tumours,Lymphomas, Granulocytic Leukaemia

**Published:** 1973-06

**Authors:** M. B. Duthie, I. D. H. Todd


*Br. J. Cancer* (1973) **27**, 485

### *Book Review*

**Thyroid Tumours, Lymphomas, Granulocytic Leukaemia.** Ed. M. Fiorentino, R. Vangelista and E. Grigoletto. (Proceedings of the 2nd Padua Seminar on Clinical Oncology, Padua, October 1972). Padua: Piccin. 210 pp. Price Lira 8000.

In this edition there are 9 papers covering various aspects of thyroid tumours (mainly cancers), 5 papers concerned with the possible eradication of chronic granulocytic leukaemia, and 7 papers on malignant lymphoma.

The first section on thyroid tumours includes a comprehensive review of the epidemiology and aetiology by Cascinelli and Balzarini who give their assessment of the conflicting evidence from many countries. After an outline of the general treatment planning of thyroid cancer, there follow papers on the technical aspects of surgery. In analysing his results of radiotherapy, Tubiana suggests that some failures may be due to too low a tumour dose, or to too small a treatment volume. Vangelista and his colleagues discuss the radioiodine treatment of thyroid cancer but only 13 of the patients treated had disseminated disease. Scandellari concludes that whilst surgery may be the treatment of choice for toxic autonomous adenoma, it can also be controlled with radioiodine.

The second section, on chronic granulocytic leukaemia, dwells on problems of neutrophil kinetics. Krakoff and his colleagues outline their radical, but so far rather unpromising, treatment protocol and Galton describes a small group of patients in whom the Ph. positive cells appear to be sufficiently sensitive to busulphan to raise the possibility of total eradication. Marmont and Damasio present their treatment of blastic metamorphosis with corticosteroids and vincristine.

The third section adds to our store of information on such current problems as the value of laparotomy (and splenectomy) in Hodgkin's disease, the results of multiple drug therapy, and the possible advantages of combining multiple drug therapy with radical radiotherapy in this condition.

Being the account of a seminar this book should not be regarded as in any way definitive or comprehensive, but it does draw attention to some current problems and will be of value to the postgraduate student.

M. B. Duthie
I. D. H. Todd